# Supplementary material for: Novel Poly-Arginine Peptide R18D Reduces α-Synuclein Aggregation and Uptake of α-Synuclein Seeds in Cortical Neurons
Source: Biomedicines. 2025 Jan 7;13(1):122. doi: 10.3390/biomedicines13010122 (PMC11763338; doi:10.3390/biomedicines13010122)
Supplement: Supplementary file 1 [file biomedicines-13-00122-s001.zip › biomedicines-3385248-supplementary materials.pdf]

biomedicines

MDPI

Supplementary Materials

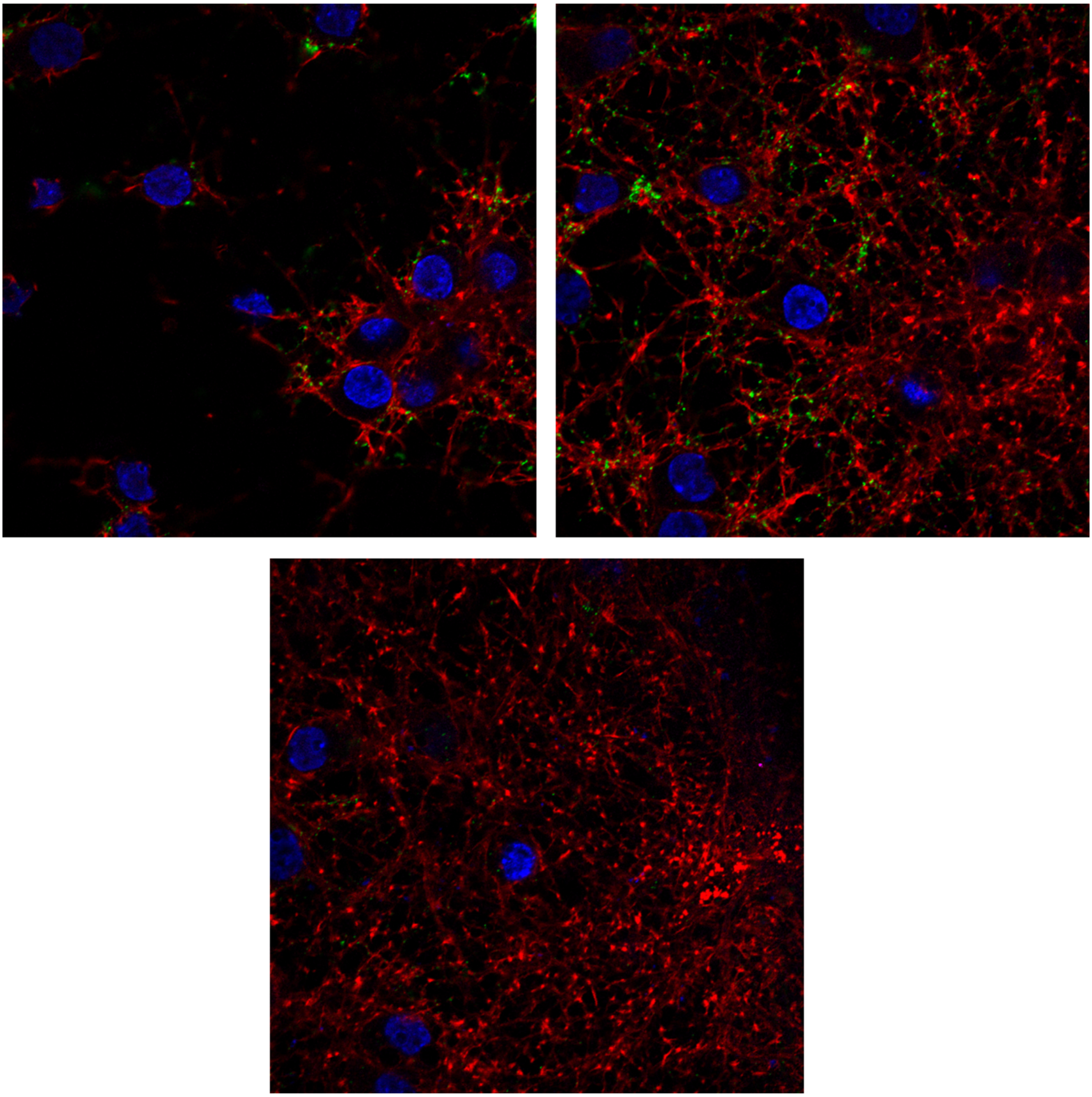

Supplementary Figure S1. Uncropped images from Figure 5B.

 https://doi.org/10.3390/biomedicines13010122

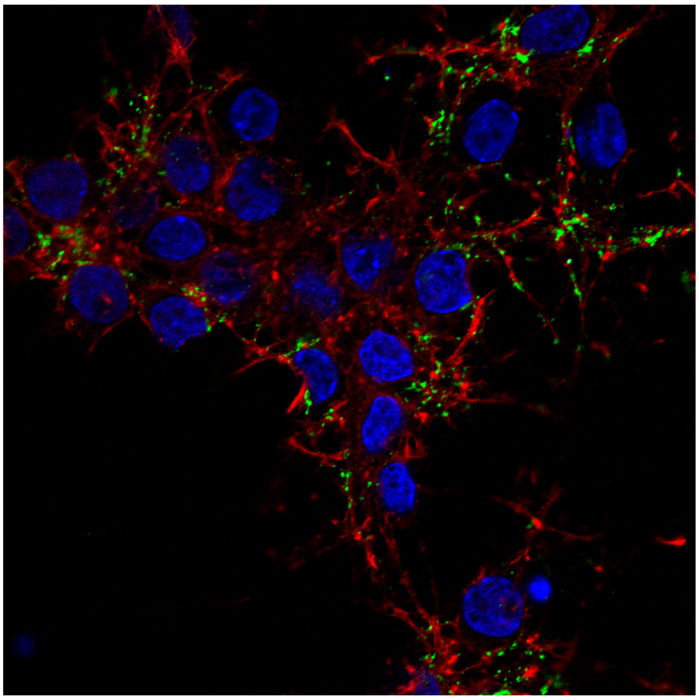

Supplementary Figure S2. Uncropped images from Figure 5D.

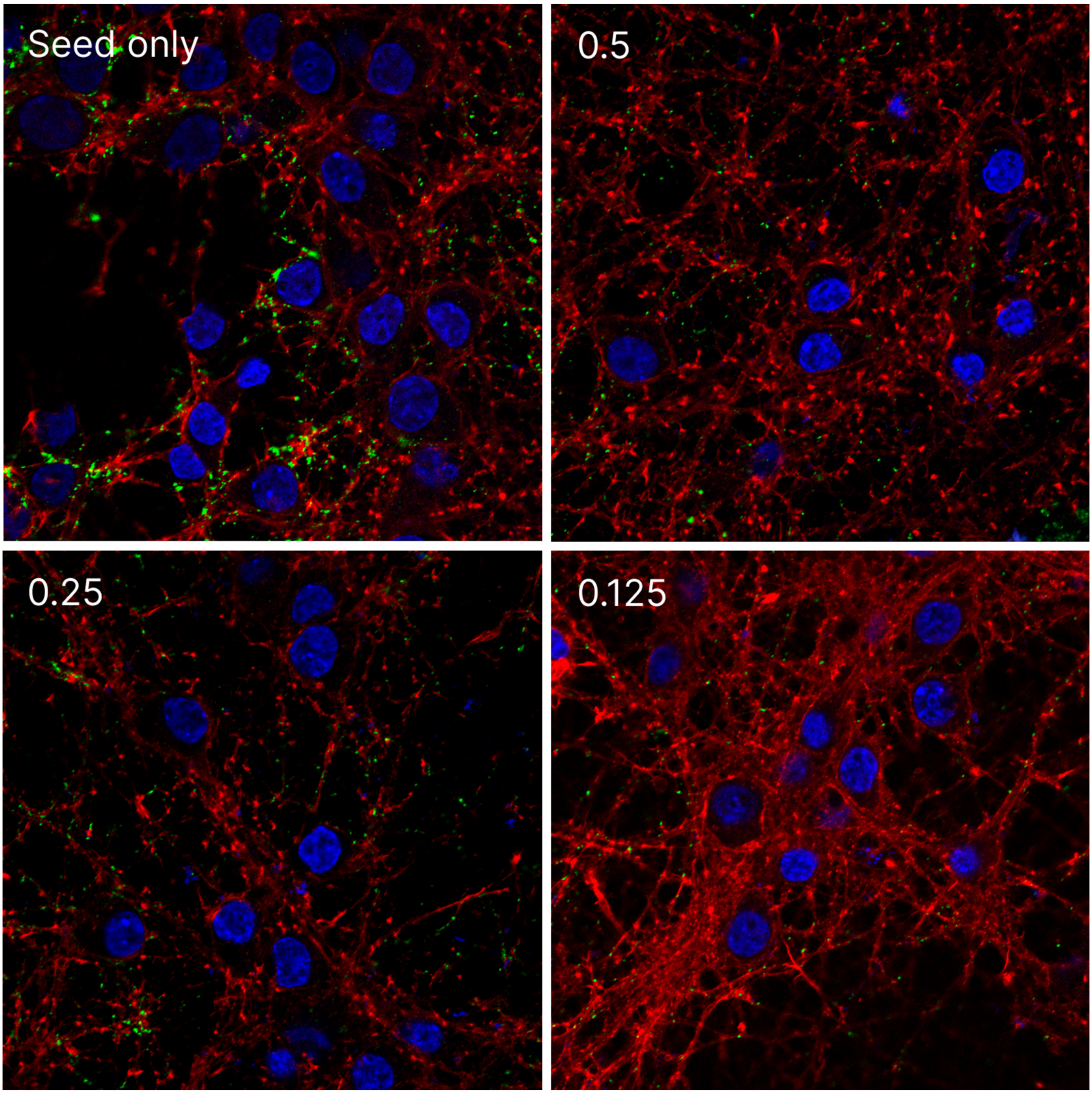

Supplementary Figure S3. Uncropped images from Figure 8.
